# **Genetic and molecular aspects of** ***Helicobacter*** ***pylori* in gastritis, pre- cancerous conditions and gastric adenocrcinoma **

**Published:** 2015

**Authors:** Mohammad Shadifar, Ramin Ataee, Amin Ataie, Ali Morad Heydari Gorgi, Nafiseh Nasri Nasrabadi, Somayyeh Nouri

**Affiliations:** 1 *Institute Pasteur of Iran, Amol research center, Amol Iran*; 2 *Pharmaceutical Sciences Research Center, Hemoglobinopathy Institute, Mazandaran University of Medical Sciences, Sari Iran*; 3 *Thalassemia Research Center, Hemoglobinopathy Institute, Mazandaran University of Medical Sciences, Sari Iran*; 4 *Department of Physiology and pharmacology, Babol University of medical sciences, Babol, Iran*; 5 *Mazandaran University of Medical sciences, EDC, Sari, Iran*; 6 *Pharmaceutical Sciences Research Center, Islamic Azad University, Tehran Iran*

**Keywords:** *Helicobacter pylori*, Apoptosis, Gastritis, P53, C-Myc

## Abstract

* Gastric adenocarcinoma* is one of the most common malignancies worldwide. Many ethological causes have been introduced among which *helicobacter pylori*, as a gram-negative bacterium has been considered as an important pathological facilitating factor. This agent is also associated with different digestive diseases, such as gastritis, peptic ulcer, and mucosa-associated lymphoid tissue lymphoma. Recently, scientists have been described some molecular aspects that show the role of some apoptotic genes and proteins; for example: P53, Bcl2, C-Myc and Rb-suppressor systems in the *H. pylori* pathogenesis. Also the relationship between nitric oxide (NOSi genotype) with *H. pylori* infection has been shown. The aim of this mini-review is to explain better these genetically aspects of *H.pylori* pathogenesis.

## Introduction

*Helicobacter pylori *is a gram-negative bacterium associated with different digestive diseases, such as gastritis, peptic ulcer, mucosa-associated lymphoid tissue lymphoma, and gastric cancer (1-3). While the infection usually starts in infancy or early childhood, there is a long latency period, and cancers are clinically diagnosed four or more decades later. During this period, a prolonged precancerous process takes place, represented by a “cascade” of events with the following well-characterized, sequential histopathological stages: chronic active non-atrophic gastritis, multifocal atrophic gastritis, intestinal metaplasia(complete, then incomplete), dysplasia, and invasive carcinoma (2, 3). At present, several diagnostic assays for *H. pylori *detection are available (2, 3). Invasive methods requiring gastric endoscopy include rapid urease testing, culture, histology, and molecular diagnostics. Non-invasive approaches include fecal antigen detection, serologic testing, and urea breath testing. During recent years, non-invasive methods are more in consider; however, no information (e.g., on resistance against antibiotics) on resistance against antibiotics can yet be obtained with these tests. Clarithromycin is the important first-line treatment option for *H. pylori *infection. Although clarithromycin is widely used as an antimicrobial therapy, the prevalence of clarithromycin-resistant *H. pylori *strains is increasing continuously.

**Figure 1:**
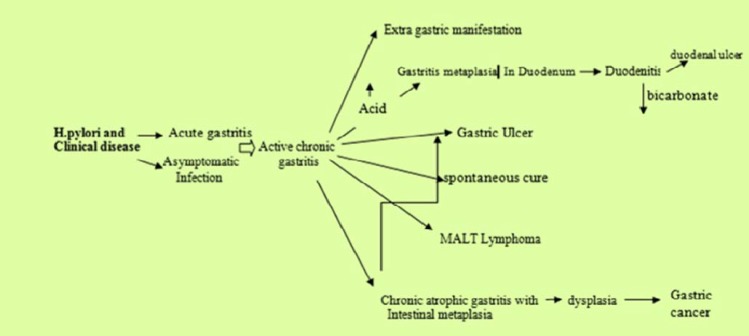
*Helicobacter pylori* and possible outcomes of infection (43)

*Helicobacter pylori *can be responsible for many gastroduodenal diseases, including acute gastritis, atrophic gastritis, intestinal metaplasia, peptic ulcer and other disorders. According to many studies, although there is a close association between gastric cancer and *H.pylori*, there have been only few studies that report gastric carcinogenesis associated with chronic *H.pylori *infection (1, 2). *H.pylori *is a class 1 gastric carcinogen. However, it is unclear whether *H.pylori *affects molecular alterations in chronic gastritis. It is well known that only a few of *H.pylori* positive patients with chronic gastritis go towards to gastric cancer, and so this relationship is not completely understood (2). Nardone et al. (3) had shown that gastric carcinogenesis is a multistep process progressing from chronic gastritis through glandular atrophy, metaplasia, and dysplasia. Acquired genomic instability, generally precedes neoplastic clonal expansion. *H.pylori *damages stimulate gastric cell proliferation which leads to mucosal repair, but which can also induce cellular DNA damage. The most frequent epiphenomenon of DNA alteration is activation of oncogenes and/or mutation of oncosuppressor genes. The role of these genes has been studied in gastric carcinogenesis, but their interrelation with *H.pylori *infection has yet to be defined. Figure 1 shows an association between *H.pylori* infection and some clinical and pathological aspects of gastrointestinal disorders. 

***H.pylori*** **Virulence genes**

 Several *H. pylori* virulence genes, as cytotoxin associated gene A (cagA) which encodes a protein that enhances the virulence of the bacterium by increasing cytokine production of the host cell have been identified. The presence of cagA is associated with more severe clinical outcomes of gastro- duodenal diseases (4). Another virulence gene is the vacuolating cytotoxin gene (vacA) that induces vacuolation in epithelial cells leading to cell damage. Researchers identified the iceA gene, which exists as two subtypes including: iceA1 and iceA2. The function of iceA1 is similar to that of type II restriction endonuclease. Many studies have demonstrated the distribution and association between *H.pylori* virulence genes and the severity of gastroduodenal diseases. However, the results are inconsistent among different geographic regions (4).

**Expression of c-Myc and p53 genes in gastric adenocarcinoma tissues and its' relation with** ***H.pylori*** **infections.**

 In some studies, it was found that the large number of chronic gastritis cases was associated with *H.pylori*. (10). In *H.pylori *(+) cases, the majority of them showed active neutrophilic infiltrate, lymphocytic infiltrate with occasional lymphoid follicles, atrophy, intestinal metaplasia and dysplasia.

 C-Myc and p53 expression are the most widely known markers of genomic instability (5); but in some studies, c-Myc and p53 expression have not been detected in *H.pylori *(+) cases(5, 6).

The p53 gene like the Rb gene, is a tumor suppressor gene, i.e., its activity stops the formation of tumors. If a person inherits only one functional copy of the p53 gene from their parents, they are predisposed to cancer and usually develop several independent tumors in a variety of tissues in early adulthood. 

This condition is rare, and known as Li-Fraumeni syndrome. However, mutations in p53 are found in most tumor types, and so contribute to the complex network of molecular events leading to tumor formation (1).

The c-*myc* gene was discovered as the cellular homolog of the retroviral v-*my *oncogene 20 years ago. It belongs to the family of *myc* genes that includes B-*myc*, L-*myc*, N-*myc*, and s-*myc*; however, only c-*myc*, L-*myc*, and N-*myc* have neoplastic potential. Inactivation of c-*myc* in rat fibroblasts caused prolongation of cell doubling time, which suggests a central role for c-*myc* in regulating cell proliferation. The frequency of genetic alterations of c-*myc* in human cancers, has allowed estimation that approximately 70,000 U.S. cancer deaths per year are associated with changes in the c-*myc* gene and its expression. Given that c-*myc *may contribute to one-seventh of cancer deaths, recent studies have been directed toward understanding the function of the c-Myc protein in cancer biology with the hope that therapeutic insights will emerge (6).

 It is indicated that increased p53 expression is an important molecular event involved in the early stage of gastric carcinogenesis. Thus, the accumulation of this biomarker may be as determining biomarker for assessing risk of the development of gastric carcinoma. It is in parallel with similar other studies' (1, 9, 10) who have reported that *H.pylori*-positive gastritis especially that accompanied with intestinal metaplasia showed a higher p53 expression. Unlike Anagnostopoulos et al.s' idea (11) about expression of p53 only in cases with high grade dysplasia as late event during the development of gastric cancer and not in pre-dysplastic stages but some studies confirmed the relationship between atrophic or metaplastic gastric mucosa with *H.pylori *infection and p53 expression.

 Marinone et al. (12) data indicated that irreversible genetic changes in the p53 protein have not yet occurred in non-neoplastic gastric mucosa with metaplasia and *H.pylori *related chronic gastritis and concluded that the increase in p53 levels is due to an increased production of the wild-type protein probably related to an inflammatory response induced by *H.pylori* infection.

In some works, *H.pylori *(-) cases were completely negative for c-Myc expression while *H.pylori *infection was associated with positive expression of c-Mycs (8). However, some researchers have found that there is no evidence of expression of c-Myc in any gastritis sample (13). c-Myc has been reported to be increased in *H.pylori*-associated gastritis which is associated with an increased cell proliferation (14, 15). It was also found that in *H.pylori *associated gastritis, there is down regulation of p27 and up-regulation of c-Myc which lead to increased cell proliferation. In addition, it was found that *H.pylori *may influence both telomerase activity and c-Myc expression in chronic atrophic gastritis (13-15).

Zhan et al. (16) showed that expression of c-Myc was significantly higher in carcinoma than that in dysplasia and metaplasia. Also the expression of c-Myc in metaplastic cases and dysplasia with *H.pylori *infection was significantly higher than those cases without infection. In this study, it was revealed that c-Myc was completely negative in cases without *H.pylori *infection. Therefore, this study supports the others who concluded that *H.pylori *infection can cause serious imbalance between cell proliferation and apoptosis in the precancerous lesions which lead to gastric carcinogenesis.

After eradication treatment, decreasing in neutrophilic and lymphocytic infiltration and in the same case in grade of the inflammatory infiltrate, was found. The number and the grade of atrophy, intestinal metaplasia and dysplasia were also significantly decreased.

 Some findings suggest that inflammatory infiltration plays a critical role in histological findings. On the other hand, some studies (7) revealed no changes in intestinal metaplasia and atrophy after *H.pylori *eradication. While others(17) reported that *H.pylori *eradication does not reduce the histologic metaplasia score, but changes the cellular phenotype of metaplasia. This change of phenotype may be an important factor in reduction of cancer incidence after eradication of *H.pylori*.

 In some studies, *H.pylori *eradication led to a significant reduction in p53 expression. The number of p53-positive patients and the grade of positivity were significantly decreased. Masaaki et al. (1) described a reduction in the expression of p53 after eradication. They reported that *H.pylori *eradication reduced gastritis activity, atrophy, and complete metaplasia, accompanied by the disappearance of genomic instability markers (1).

**Role of bcl2, bax and p53-RB tumor suppressor system in pathogenesis of** ***H.*** ***p*** ***ylori***

 Gastric cancer occurs after a multi-step process of alterations in oncogenes, tumor-suppressor genes, cell-adhesion molecules, telomerase as well as genetic instability at several microsatellite loci. Studies have demonstrated that *H.pylori* infection is closely associated with these abnormal alterations.

 Konturek’s study (18) showed that *H.pylori *induced apoptosis in gastric mucosa through up-regulation of Bax and bcl-2 expression (19). The bcl-2 gene family plays an important role in regulating of apoptosis. Bcl-2 family proteins regulate and contribute to programmed cell death or apoptosis. It is a large protein family and all members contain at least one of four BH (bcl-2 homology) domains. Certain members such as Bcl-2, Bcl-xl and Mcl1 are anti-apoptotic, whilst others are pro-apoptotic. The pro-apoptotic group of Bcl-2 proteins can be further sub-divided into the structurally diverse 'BH3' only proteins (e.g. Bid, Noxa, Puma and Bad) and the multidomain proteins that share BH1 to 3 (e.g. Bax and Bak). Most Bcl-2 family members contain a C-terminal transmembrane domain that functions to target these proteins to the outer mitochondrial and other intracellular membranes (1,19).

 In some studies (16), *H.pylori *enhanced the expression of c-myc and bcl-2 significantly in intestinal metaplasia type 3(IMIII) (19).

 The c-myc protein is a critical component for the control of normal cell growth, but the altered c-myc activity by translocation, amplification, overexpression, and mutation is widespread in tumor cells and is important for multi-step carcinogenesis(19, 20). C-myc is a strong inducer of proliferation and is believed to be critical for the oncogenic properties. Some studies showed that the abnormal c-myc expression derived cells inappropriately through the cell cycle, leading to uncontrolled proliferation, a characteristic of neoplastic cells.

 From some studies, it was found that *H.pylori *infection caused a higher proliferation and a lower apoptosis in intestinal metaplasia type 3(IMIII) than in CG (chronic gastritis) or IMI-II (intestinal metaplasia type 1 and 2) so could accelerate cell proliferation which made a good chance for all kinds of genes and proteins to mutate or overexpress, presumably heightening the genetic instability consistent with the development of carcinoma (21, 22).

 Mutations in the tumor suppressor genes p53 and Rb are common events in human cancers that exert their control on the cell cycle at the G1-S phase transition through independent but interconnected pathways (23, 24). Williams’s studies showed that germ-line mutation in p53 and Rb might have cooperative tumorigenic effects in mice (25). It has been generally accepted that p53 and Rb tumor-suppressor system, including p53, Rb, p16, p15, p14 and p21waf1(26, 27), have important roles in carcinogenesis. Some studies showed this phenomenon that NO (nitric oxide) generated by *H.pylori *caused p53 mutation at the spot C:G to A:T. At the same time p53 was found mutated at the same spot in IM, Dys and GC (intestinal metaplasia, dysplasia and gastric cancer), thus *H.pylori* probably caused p53 mutation. In a study (16), it was found that the expressions of p53-Rb were continually enhanced from chronic gastritis (ratio:3/42) to gastric cancer (ratio:76/84) and in the DysIII (Dysplasia phase 3) and GC (gastric chronic) which had a close association with *H.pylori *(18).

 Chen (26) reported that the mRNA levels of p53-Rb in gastric cancer were significantly lower than those in their non-cancerous tissues using quantitative analysis method. Some studies also showed that c-myc drove initial proliferation and subsequent differentiation, concomitant with the activation of the p53G2 checkpoint and also demonstrated that inactivation of the p53-Rb pathway is required for immortalization through overexpression of Myc (27, 28). 

**Activity of Tolmerase enzyme and its role in progression of gastric tumor and H. Pylori pathogenesis**

 Telomerase is a unique ribonucleoprotein enzyme that is responsible for adding the telomic repeats onto the 3’ end of chromosomes and composed of a catalytic protein submit (hTERT, for human telomerase reverse transcription) and a template RNA (TR), and hTERT is the rate-limiting enzyme in the telomerase complex (29, 30). The enzyme telomerase allows for replacement of short bits of DNA known as telomeres, which are otherwise shortened when a cell divides via mitosis. In normal circumstances, without the presence of telomerase, if a cell divides recursively, at some point all the progeny will reach their Hayflick limit, which is believed to be between 50–70 cell divisions until the cells become senescent and cell division stops. With the presence of telomerase, each dividing cell can replace the lost bit of DNA, and any single cell can then divide unbounded. While this unbounded growth property has excited many researchers, caution is warranted in exploiting this property, as exactly this same unbounded growth is a crucial step in enabling cancerous growth (31,32).

From the above explanations, it is apparent that understanding of the association between *H.pylori *infection with some genes (for example p53, Rb) as well as telomerase can contribute to the elucidation of the mechanisms that regulate the development of gastric cancer. 

 Telomerase activity has been found in 85-90% of all human cancers, but not in adjacent normal cells (28). It has thus been hypothesized that for a cancer cell to undergo sustained proliferation beyond the limits of cell senescence, it must reactivate telomerase or an alternative mechanism in order to maintain telomeres. This makes telomerase as a target not only for the novel etiology agent but also a mark for cancer diagnosis. Many studies showed that telomerase activity was higher in cancers than in non-cancerous tissues and higher in intestinal metaplasia (IM) than in chronic gastritis (CG) (29,33,34). The association between *H.pylori *infection and telomerase activity is still controversial. Suzuki’ study (28) indicated that hTERT mRNA which was expressed in precancerous lesions and gastric cancer could be induced at an early stage of gastric carcinogenesis, but it was not correlated with *H.pylori*.

 Kuniyasu (32) found that *H.pylori *evidently caused the release of reactive oxygen species (ROMs) and reactive nitrogen species (NO) which might be strong triggers for “stem cell” hyperplasic in intestinal metaplasia followed by telomere reduction and increased telomerase activity as well as hTERT overexpression.

 Jing Lan et al. found in chronic gastritis (CG), hTERT expression was significantly higher in infectious group (ratio:47/48) than in non-infectious group (ratio:30/36) but had no association have been observed in Dys or IM(intestinal dysplasia or metaplasia), maybe because there exist two different genetic pathways and two histological types: intestinal-type and diffuse-type gastric cancer. Some studies showed c-myc could stimulate expression of hTERT and thereby enhance telomerase activity which was an important step in carcinogenesis (33-38). In J lan et al. (18) study c-myc had an association with hTERT in Dys and GC (dysplasia and chronic gastritis), which suggests that *H.pylori *can stimulate telomerase directly or indirectly by the overexpression of c-myc. J Lan did not find an association between hTERT and p53-Rb or bcl-2 (18).

**Inducible nitric oxide synthetase genotype as a risk factor for** ***H.pylori*** **pathogenesis**

 Rafiei *et al* ,Shen et al and Xu (38-41) recently has shown that “Inducible nitric oxide synthetase genotype and *H. pylori *infection increase gastric cancer risk”. In Rafiei *et al's* experiment, they demonstrated the possible association between inducible nitric oxide synthase (*iNOS*) genotype and gastric cancer among the 329 patients from northern Iran. They found a clear association between the presence of C150T polymorphism of the *iNOS *gene and the presence of gastric cancer in *H. pylori* infected patients. In addition, they found an overall frequency of the 150T allele of approximately 25% of the individuals (both controls and cases) tested (38). Interestingly the 3’ end of this 288 bp sequence (supposedly containing the binding site for the reverse primer) differs considerably from all but one of the other *iNOS* sequences in the database; i.e., an 288 *iNOS* sequence with accession no X85772.1. This closely homologous sequence (X85772.1) differs only in the C150T position and was submitted to the database by Xu *et al *(42).

## Conclusions

According to many studies about the role of *helicobacter pylori* in gastric cancer and gastritis, we can conclude that *H*.*pylori* infection has multifunctional mechanisms as inducing proliferation of cells and inducing some oncogenes or suppress some inhibitor genes which can develop chronic gastritis and intestinal metaplasia or dysplasia towards adenocarcinoma. Among these genes and proteins, role of P53, Bax, Bcl2, C-Myc and Rb-supressor almost have been demonstrated. It was also revealed that *H.pylori* metaplasia and dysplasia can change in telomerase activity and the telomere contents, which bring dysplasic situations towards cancer. Also in recent years, the relationship between C150T polymorphism of NOSi gene has been found. Although, the etiology of gastric adenocarcinoma and its' relationship with H.pylori has not been revealed exactly, but this relation is in scope of pharmatherapeutics researches, and yet H.pylori eradication is known as a goal for gastric cancer prevention.
